# Sperm parameters, protamine deficiency, and apoptosis in total globozoospermia

**Published:** 2015-08

**Authors:** Jalal Ghasemzadeh, Ali Reza Talebi, Mohammad Ali Khalili, Farzaneh Fesahat, Iman Halvaei, Ali Nabi, Sareh Ashourzadeh

**Affiliations:** 1 *International Campus, Shahid Sadoughi University of Medical Sciences, Yazd, Iran.*; 2 *Research and Clinical Center for Infertility, Shahid Sadoughi University of Medical Sciences, Yazd, Iran.*; 3 *Afzalipour Clinical Center for Infertility, Afzalipour Hospital, Kerman University of Medical Sciences, Kerman, Iran.*

**Keywords:** *Male infertility*, *DNA damage*, *Sperm protamine*, *Apoptosis*

## Abstract

**Background:**

Globozoospermia is a severe form of teratozoospermia (incidence < 0.1%) in infertile men that is characterized by round headed sperm and acrosomeless in semen.

**Objective:**

To compare the semen parameters, protamine deficiency, and apoptosis in ejaculated spermatozoa between globozoospermic and normozoospermic men.

**Materials and Methods:**

Thirty six semen samples were divided into two groups including 15 infertile men with total globozoospermic (> 90% round-headed sperm) and 21 healthy donors with normal spermograms as controls. Semen analysis was performed according to World Health Organization criteria (2010). Sperm protamine deficiency was assessed using Chromomycin A3 (CMA3) staining and the rate of apoptotic spermatozoa was evaluated with TUNEL assay.

**Results:**

Sperm concentration, motility, and normal morphology in globozoospermic men were significantly decreased compared with controls (p<0.05). The rate of CMA3-reacted spermatozoa (CMA3+) in globozoospermic men was higher than controls (65.93 ± 11.77 vs. 21.24 ± 7.37, respectively, p<0.0001). The rate of apoptotic spermatozoa (TUNEL positive) were significantly increased in globozoospermic cases with respect to the controls (17.60 ± 10.72 and 5.95 ± 3.02, respectively, p<0.0001). There was no significant correlation between sperm protamine deficiency and apoptosis in globozoospermic men.

**Conclusion:**

Globozoospermic samples contain a higher proportion of spermatozoa with abnormal chromatin packaging and DNA fragmentation than normozoospermic samples. Therefore, in addition to absence of acrosome in the spermatozoa of globozoospermic patients, the high percentage of spermatozoa with immature chromatin and apoptotic marker may be considered as the other etiologies of infertility in these patients.

## Introduction

Globozoospermia is a severe form of teratozoospermia with very low incidence (< 0.1%) in infertile patients. Globozoospermia is classified as total owing to the absence of any sperm with acrosome (type 1) and partial in that the ratio of round-headed spermatozoa is less than 100% (i.e., 20-90%) and acrosomal vesicles are present in the rest of the spermatozoa (type 2) (1). The pathogenesis of globozoospermia syndrome is not well known. The most important morphological characteristics of these spermatozoa are including round headed, acrosomeless, round nucleus, no post-acrosomal sheath and coiled tail. Despite the persistence of other normal sperm parameters such as count, density and motility, globozoospermia is considered as one of the important etiologies of male infertility with very low ART success rates (2-4). It is shown that globozoospermia as well as other abnormal morphologic forms of human spermatozoa is associated with DNA strand breaks and abnormal chromatin structures. Some recent studies have reported high amounts of disrupted replacement of nuclear histones by sperm specific protamines (5), increased P1/P2 ratio, DNA strand breaks and other sperm chromatin abnormalities (6-8) in sperm cells of globozoospermic patients. Although, some studies have shown no significant increase in DNA denaturation or sperm DNA fragmentation in globozoospermic cases compared fertile men (6-7, 9), Sutovsky et al in 2001 with the introduction of SUTI (sperm-ubiquitin tag immunoassay) technique showed that globozoospermic are highly ubiquitinated that indicated their damaged DNA (10). In fact, these results suggest that abnormal globozoospermic spermatozoa can be carrier of abnormal chromatin as a possible source of DNA fragmentation.

Among the different hypothesis for the origin of DNA fragmentation of sperm cell, abortive apoptosis is considered as one of the main mechanisms that could explain these results. According to this hypothesis sperm with abnormal chromatin and apoptotic markers are presented in the ejaculate due to inefficient sperm removing by Sertoli cells (11, 12). Also, the chromatin with abnormal compaction can be more sensitive to external stresses such as reactive oxygen species that induce DNA fragmentation (13). The round-headed spermatozoa may be containing an abnormal chromatin with DNA strand breaks. It can cause the early pregnancies loss or increase the risk of birth malformations, cancer, and genetic disorders in newborns (14). Therefore, the evaluation of chromatin structure status and sperm DNA in globozoospermic patients should be considered as one of the basic laboratory assessments. As it is mentioned above, there are few studies indicating the changes of sperm chromatin structure in these patients, but, there are controversies in the results (6, 9-10, 15). The main reason for different reports may be the low frequency of globozoospermia among from infertile couples (15). In fact, most studies are presented as case reports using low number of semen samples to evaluate the semen parameters and sperm chromatin structure (2, 6, 9, 15). Therefore, the aim of this study was to compare the semen parameters, protamine deficiency, and apoptosis in ejaculated spermatozoa between total globozoospermic and normozoospermic men.

## Materials and methods

**Patient’s selection**

In this case-control study, 36 semen samples of fertile and infertile men referred to andrology laboratory of Yazd Research and Clinical Center for Infertility from June 2010 to November 2014 were evaluated in two groups. The case group consisted of 15 infertile men with total globozoospermia (with more than %90 round headed spermatozoa) and the control group consisted of 21 fertile men with normal semen parameters and whose partners had successful pregnancies within the last one year and their samples were taken before vasectomy. A complete evaluation in globozoospermic patients including physical examinations, smoking, and cytogenetic, immunological and reproductive hormonal assays were done. Men with heavy smoking, drug and alcohol abuse, varicocele and age ≥45 years which may impact the sperm DNA integrity were excluded. This study was approved by institutional review board of Yazd Research and Clinical Center for Infertility and informs consent forms signed by all participants.

**Sperm collection and analysis**

Semen samples were collected by masturbation after 2-5 days of sexual abstinence. Each semen sample was allowed to undergo liquefaction, and then was evaluated for sperm motility, concentration, and morphology according to World Health Organization (WHO, 2010) criteria (16). Briefly, sperm motility including progressive motility (PR), non-progressive motility (NP), and immotile (IM) were assessed manually by phase-contrast microscopy (Zeiss, Axiostar plus, Germany) at X400 magnification. Papanicolaou staining was applied to evaluate morphological abnormalities and at least 200 sperm cells were examined per slide. Sperm count was assessed by Makler counting chamber (Sefi Medical Co., Haifa, Israel) (17). All analyses were done twice by one experienced laboratory technician blinded to the study.

**Sperm DNA integrity tests**

For the evaluation of sperm chromatin structure, two tests were used including; TUNEL assay for sperm apoptosis detection and chromomycin A3 (CMA3) for sperm protamine deficiency.

**Terminal** **deoxynucleotidyl** **transferase-mediated dUTP nick-end labeling (TUNEL) assay**

**Figure 1:**
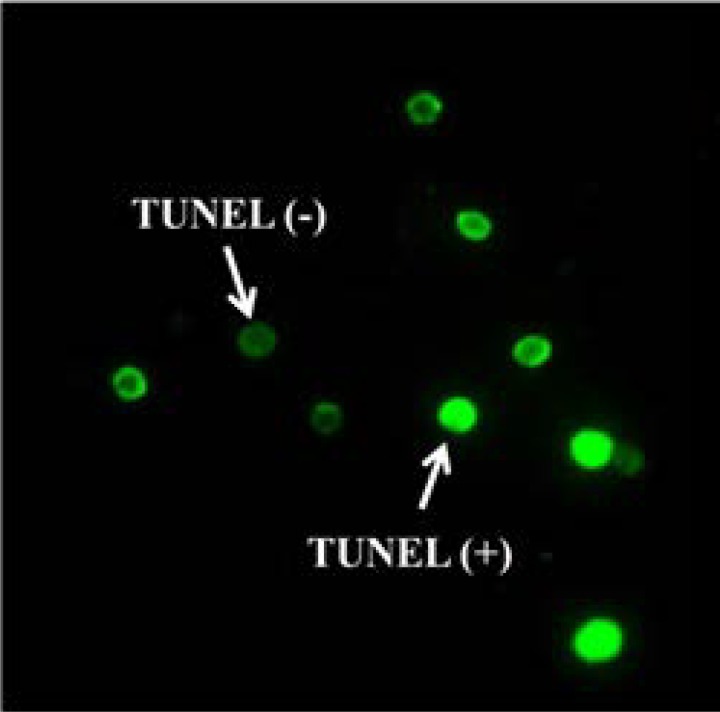
TUNEL assay for detection of sperm apoptosis. Under fluorescent microscopy normal DNA (non-apoptotic sperm) was is seen light green and damaged DNA (apoptotic sperm) was is seen bright green (× 100 eyepiece magnification).

The percentage of apoptotic spermatozoa in each sample was determined by TUNEL assay by In Situ Cell Death Detection Kit (Roche Diagnostics GmbH, Mannheim, Germany) (18). In this method, the smeared samples were fixed with 4% paraformaldehyde in PBS (Phosphate buffer saline) for 15 minutes at room temperature. After washing with PBS, the samples were incubated with 0.3% H_2_O_2_ in methanol for 1 hour to quench endogenous peroxidase activity. The cell permeability was done with 0.1% Triton X-100 (Sigma Aldrich Company ,St. Louis, USA) at 4^◦^C for 5 minutes, and then incubated with the TUNEL reaction mixture (50 µl) in a humidified chamber and dark room at 37^◦^C for 1 hour. After washing in PBS, they stained with 50 µl converter-POD (peroxidase) at 37^◦^C for 1 hour. Samples were washed in PBS and exposed to the DAB (3, 3-diaminobezidine tetrahydrochloride) (Roche Applied Science, Mannheim, Germany) substrate solution for color development in a dark chamber at room temperature for 10 minutes. Finally, the samples were dehydrated in ethanol, cleared by xylene, mounted by DPX (Shandon, Thermo scientific, USA) and then evaluated by fluorescent microscope under X100 eyepiece magnification in each sample, at last 200 sperm nuclei were counted and repeated again. For negative controls, instead of the TUNEL reaction mixture, slides were incubated with 50 µl of label solution (without terminal transferase). The rate of spermatozoa with light green heads (TUNEL- or non-apoptotic sperm) and bright green heads (TUNEL+ or apoptotic sperm) were determined (Figure 1).

**CMA3 staining**

**Figure 2:**
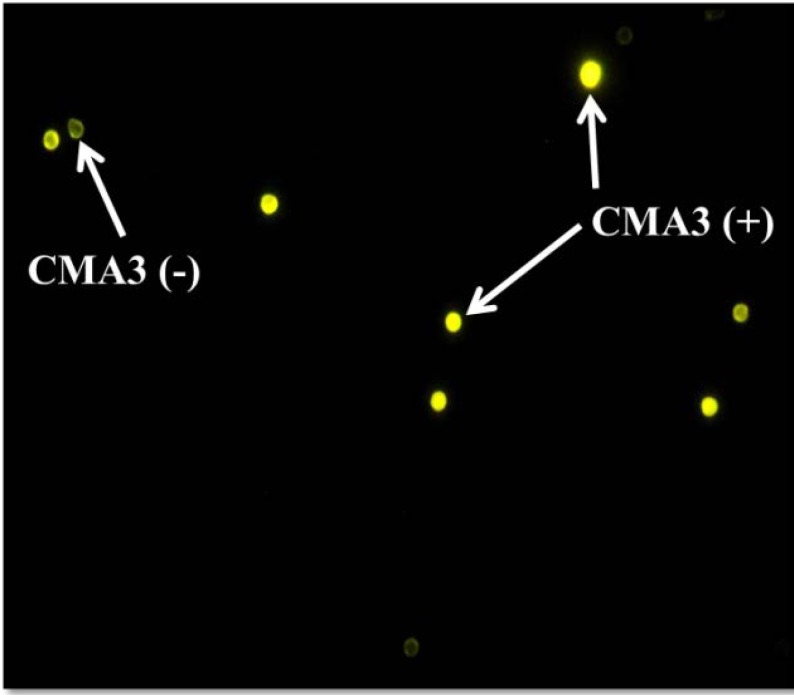
CMA3 staining for evaluation of sperm protamine deficiency. Bright yellow round-headed sperm cells (CMA3+) show protamine deficiency and yellowish green round-headed sperm cells (CMA3-) show normal protamine content (fluorescent microscopy, × 100 eyepiece magnification).

Chromomycin A3 is a fluorochrome specific for guanosine cytosine-rich sequences and is used for evaluation of the degree of protamination of sperm chromatin (19). To do this test, the air-dried smears were fixed by Carnoy’s solution (methanol/glacial acetic acid, 3:1) for 10 minutes at 4^◦^C. The slides were stained by CMA3 solution (Sigma, St Louis, MO, USA) (0.25 mg/ml in McIlvaine buffer; 7 ml citric acid, 0.1 M + 32.9 ml Na2HPO4 7H2O 0.2 M, pH 7.0 containing 10 mM MgCl2) for 10 minutes at room temperature. After washing, the slides were mounted by DPX, and then at least 200 spermatozoa were counted under florescent microscopy (BX51, Olympus, Tokyo, Japan) with a 460-nm filter and X100 eyepiece magnification. The percentage of spermatozoa with bright yellow heads (CMA3+ with protamine deficiency) and without brightness (CMA3-with normal protamine content) was evaluated in each sample (Figure 2).

**Statistical analysis**

The results were analyzed using the Statistical Package for the Social Sciences, version 20.0, SPSS Inc, Chicago, Illinois, USA. Means were reported as mean ± standard deviation. Student’s *t*-test was applied to compare the groups, and the term ‘statistically significant’ was used to denote a two-sided p-value < 0.05. The correlation between apoptosis and protamine deficiency was performed using Pearson coefficient of correlation.

## Results

The mean age of globozoospermic and fertile men were 35.40±6.20 and 34.63±2.85 years. Cases had a mean duration of 8.61 years of primary infertility. TUNEL assay and CMA3 staining results performed for detection of sperm apoptosis and protamine deficiency are shown in figure 1 and 2, respectively.

**Figure 3:**
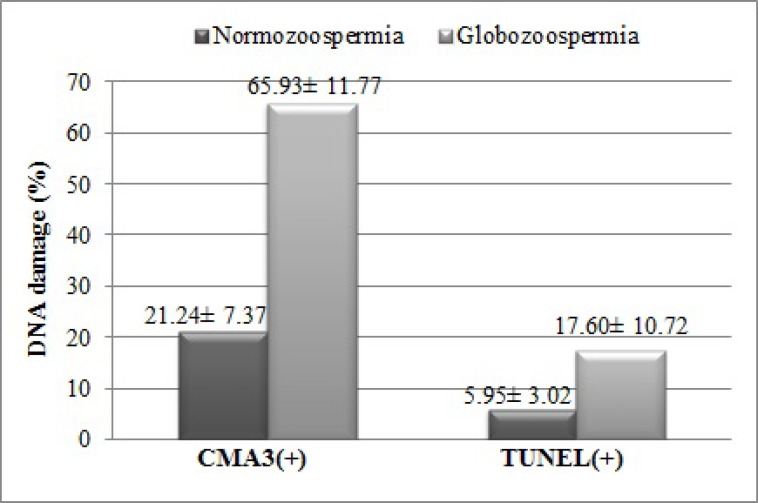
Comparison of DNA damage according to CMA3 and TUNEL tests in case and control groups

The results of sperm parameters from case and control groups are listed in table I. The normal morphology and progressive motility showed a statistically significant difference between two groups (p-value< 0.000). The percentage of spermatozoa with protamine deficiency and apoptosis were significantly higher in case group than controls (Table II, Figure 3). However, Pearson coefficient of correlation showed no correlation between sperm nuclear protamine deficiency and sperm apoptosis rate (p= 0.91, r= 0.032).

**Table I:** The results of sperm parameters in case and control groups

**Variables**	**Case group (n=15)**	**Control group (n=21)**	**p-value**
Volume (ml )	3.24 ± 1.21	3.02 ± 1.73	0.685
Concentration (×10^6^/ ml)	78.07 ± 30.26	105.14 ± 46.12	0.055
Rapid progressive (%) (grade a)	5.20 ± 7.65	25.52 ± 10.40	0.000*
Slow progressive (%) (grade b)	28.73 ± 9.02	38.05 ± 8.51	0.003*
Non-progressive motility (%) (grade c)	14.20 ± 5.15	9.86 ± 3.97	0.007*
Immotile (%) (grade d)	51.87 ±10.68	26.57 ± 6.86	0.000*
Progressive motility (%) (grade a + grade b)	33.93 ±12.91	63.57 ± 8.70	0.000*
Normal morphology (%)	0.00 ± 0.00	35.95 ± 8.98	0.000*

Data are presented as mean± SD.

* Student’s *t* test.

**Table II:** The results of sperm chromatin/ DNA evaluation in case and control groups

**Variables**	**Case group (n=15)**	**Control group (n=21)**	**p-value**
CMA3 (+)	65.93 ± 11.77	21.24 ± 7.37	0.000*
TUNEL (+)	17.60 ± 10.72	5.95 ± 3.02	0.000*

Data are presented as mean± SD.

* Student’s *t* test.

## Discussion

Globozoospermia is a rare but severe disorder causing male infertility was first described in 1971. Total globozoospermia is diagnosed by the presence of about 100% round-headed spermatozoa lacking an acrosome (20). On the other hand, these spermatozoa do not have sperm-associated oocyte-activating factor for Ca^2+^ flux that is essential for fertilization. Therefore, they unable to pass the zona pellucida and fuse to the oolemma (1, 12). In present study, semen parameters, protamine deficiency and apoptosis were compared between globozoospermic patients and fertile men. Our findings showed statistically significant differences in sperm morphology and motility between case and control groups. There was a significant decrease in rapid and slow progressive motility, progressive motility and normal morphology in case group compared with controls. Despite a slight decline in semen volume and sperm concentration in case group, it was not statistically significant. Dam et al. retrospectively investigated 72 semen samples from globozoospermic patients and their findings was precisely confirmed our results (1). Although, most other human studies suggested a decrease in normal morphology and motility like our results (1, 21), there were controversial finding in few studies about sperm motility changes in these patients (2, 9). There were two probably reasons about conflicting results with ours: one is that we analyzed semen parameters based on standard WHO (2010) that have more strict criteria compared to previous protocols (1992 and 1999).The other was significant differences in our sample size in comparison with such these studies presented as case reports.

Abnormal sperm chromatin condensation and DNA damage may have a negative effect on fertility potential. Approximately 15% of patients with male factor infertility have a normal semen analysis and a definitive diagnosis of male infertility often cannot be made as a result of routine semen analysis (22). Therefore, sperm nuclear DNA integrity in male factor infertility should be assessed in couples with globozoospermia (23). In present study we used CMA3 for detection of sperm protamine deficiency. Our results demonstrated that men with globozoospermia have higher percentage of spermatozoa with protamine deficiency in their samples than controls. This chromatin abnormality probably is persistent because of some disorders during spermiogenesis causing round-headed sperm cells in globozoospermic cases.

Nardo et al. (2002) and Taylor et al. (2010) by two separated studies on ultra-structural features of globozoospermic sperm cells expressed the low density chromatin in these patient samples (24, 25). Dam et al also got to a similar conclusion in this regard (1). Our results were in agreement with Deemeh (2007) who showed the high levels of protamine deficiency in 2 men with globozoospermia using CMA3 test (11). Recently, Vozdova and colleagues (2014) showed that a patient with globozoospermia had more CMA3 and aniline blue positive spermatozoa in comparison with fertile men which is in line with our findings. This study also proposed that poor chromatin quality of sperm may be considered as an important cause of pregnancy failure (6). According to our results, protamine deficiency was another characteristic of sperm cells from globozoospermic patients. Since, protamine deficiency and excessive histones are related to each other, spermatozoa from these patients have abnormal chromatin remodeling. Additionally, in some cases of male factor infertility, there are a high percentage of CMA3 + spermatozoa and this test may be used as a good predictor of male infertility (24, 26). Agarwal and said described count, motility, and morphology of sperm cells in infertile couples were related to extent of DNA damage. They suggested that in many cases, although the DNA damage may not prevent the in vivo fertilization of oocyte but, the resulting zygote fails to get enough growth potential leads to early pregnancy losses (27).

One of the main goals of our study was to investigate the rates of sperm apoptosis in globozoospermic patients using TUNEL assay. We showed that these cases have more spermatozoa apoptosis than controls. Perrin et al. showed increased levels of DNA fragmentation in 6 men with globozoospermia when compared with fertile men using TUNEL assay (28). At one case report study, similar results were detected on increased levels of DNA fragmentation and apoptosis in sperm sample of globozoospermic patient (8). In opposite to our finding, few case reports were expressed no significant change in rates of sperm DNA fragmentation of globozoospermia (29). It is clear that the main reason for this difference can be different sample sizes. Although, the apoptosis is considered as the main cause of DNA strand breaks in human spermatozoa, but DNA fragmentation in mature spermatozoa have other origins beside apoptosis. Abnormal chromatin packaging during spermiogenesis and oxidative stress are also considered as the other sources of sperm DNA damage (30). There are several studies indicating the relationship between sperm chromatin anomalies and sperm DNA fragmentation which is a critical step in apoptosis (13, 18, 31). We showed that both sperm protamine deficiency and apoptosis are seen in semen samples of globozoospermic patients. But there was not any statistically significant correlation between these parameters (p=0.91). It may be due to the other DNA damages and apoptosis mechanisms in globozoospermia men. 

## Conclusion

Our results showed that globozoospermic samples contain a higher proportion of spermatozoa with abnormal chromatin packaging and DNA fragmentation compare with fertile men. In fact, in addition to absence of acrosome and oocyte activation factor in the spermatozoa of globozoospermic patients, the high percentage of spermatozoa with immature chromatin and apoptotic marker may be considered as the other etiologies of infertility in these patients.

## Note

***This article extracted from M.Sc. thesis. (Jalal Ghasemzadeh)***
